# Detection of *MSH*2 Gene Methylation in Extramammary Paget's Disease by Methylation-Sensitive High-Resolution Melting Analysis

**DOI:** 10.1155/2021/5514426

**Published:** 2021-11-01

**Authors:** Liu Dong, Yingfeng Zhu, Liting Wu, Qiaoan Zhang, Feng Xu, Xinju Zhang, Xiao Xu, Yiting Tang, Guoqiang Ren, Zhihua Kang, Ming Guan

**Affiliations:** ^1^Department of Laboratory Medicine, Huashan Hospital, Shanghai Medical School, Fudan University, Shanghai, China; ^2^Department of Pathology, Huashan Hospital North, Fudan University, Shanghai, China; ^3^Department of Laboratory Medicine, Shidong Hospital of Yangpu District, 999 Shiguang Road, Shanghai, China; ^4^Department of Dermatology, Huashan Hospital, Fudan University, Shanghai, China; ^5^Central Laboratory, Huashan Hospital, Fudan University, Shanghai, China; ^6^Rutgers Cancer Institute of New Jersey, Rutgers University, New Brunswick, NJ, USA

## Abstract

**Background:**

Extramammary Paget's disease (EMPD) is a rare skin tumor. Hypermethylation in the *MSH*2 promoter resulting in the downregulation of its protein expression shows a high detection rate in EMPD tumor tissue, which indicates that the methylation of *MSH*2 may play an important role in the pathogenesis of EMPD.

**Objective:**

This study aims to establish a rapid analysis strategy based on the methylation-sensitive high-resolution melting curve (MS-HRM) to detect the methylation level of the *MSH*2 promoter.

**Methods:**

With the use of universal methylated human DNA products, we established the MS-HRM standard curve to quantitatively detect the methylation level of the *MSH*2 promoter. Then, all 57 EMPD tumor DNA samples were analyzed. Pyrosequencing assay was also carried out to test the accuracy and efficacy of MS-HRM. Besides, a total of 54 human normal and other cancerous tissues were included in this study to test the reliability and versatility of the MS-HRM standard curve.

**Results:**

In this study, by using the established MS-HRM, we found that 96.5% (55/57) EMPD tumor samples had varying methylation levels in the *MSH*2 promoter ranging from 0% to 30%. Then, the methylation data were compared to the results obtained from pyrosequencing, which showed a high correlation between these two techniques by Pearson's correlation (*r* = 0.9425) and Bland–Altman plots (mean difference = −0.1069) indicating that the methylation levels analyzed by MS-HRM were consistent with DNA pyrosequencing. Furthermore, in 23 normal and 31 other cancerous tissue samples, there were two colorectal cancer (CRC) tissues that tested *MSH*2 methylation positive (1% and 5%) which confirmed that our established MS-HRM can be widely applied to various types of samples.

**Conclusion:**

MS-HRM standard curve can be used for the detection of the methylation level of *MSH*2 in EMPD tumor samples and other cancerous tissues potentially, which presents a promising candidate as a quantitative assay to analyze *MSH*2 promoter methylation in routine pathological procedure.

## 1. Introduction

Extramammary Paget's disease (EMPD) is a rare intraepithelial adenocarcinoma which is most likely to appear in the skin containing apocrine sweat glands, such as anogenital and axillary regions [1]. The clinical presentation of this skin cancer is eczema-like, exhibiting pruritus, burning, or tenderness in affected lesions. Misdiagnosis as skin inflammation or eczema is frequent in most of the patients with EMPD, which probably delays the effective therapies and also leads to the high risk to develop secondary malignant tumors causing death outcomes eventually [2]. The histological feature of EMPD is the infiltration of Paget cells in skin lesions. These unique cells can be identified through cytokeratin staining such as *CK*7 and *CK*20 and some other histopathologic biomarkers [3].

So far, the pathogenesis of EMPD is largely unclear. Whether EMPD is of epidermotropic or intraepidermal origin is still in dispute [4]. Through whole-exome sequencing, Zhang et al. [4, 5] revealed the full genomic mutational profiles of EMPD, demonstrating that *KMT*2*C*, *ARID*2, and *FOXA*1 mutations were frequent in EMPD, and other driver gene mutations, such as those in *PIK*3*CA*, *KRAS*, *BRAF*, and *AKT*1, have also been reported [6].

In our previous study, it has been proved that germline mutations in mismatch repair (MMR) genes were common in EMPD patients. About 40% cases had missense mutations in key genes of MMR, such as *MSH*2, *MLH*1, *MSH*6, and *PMS*2 [7]. Reduced *MSH*2 was observed in 38.6% EMPD cases, possibly due to germline mutations or epigenetic alterations in this gene [8]. Defects in MMR caused by DNA methylation in MMR key genes, *MLH*1 in particular, account for most of the loss of MMR proteins' expression [9]. Considering the high incidence of low *MSH*2 expression in EMPD, it is meaningful to detect *MSH*2 promoter methylation in EMPD tumor tissues effectively.

DNA methylation is one of the most common epigenetic events which can alter gene expression without changes in genomic DNA sequences, thus making the detection of DNA methylation status more reliable than single analysis of protein expression [10]. Multiple techniques have been developed to detect the methylation status of specific genes such as bisulfite sequencing PCR (BSP), pyrosequencing, methylation-specific PCR (MSP), real-time quantitative methylation-specific PCR (qMSP), and methylation-sensitive high-resolution melting (MS-HRM). Both BSP and MSP are based on DNA sequencing techniques which are able to analyze DNA methylation status quantitatively at the base resolution. Despite the complexity of pyrosequencing, it is considered the gold standard for DNA methylation detection [11, 12]. MSP, qMSP, and MS-HRM are all PCR-based detection methods [13, 14].

In this study, MS-HRM technique is employed. HRM analysis can accurately distinguish single base pair changes through fluorescence monitoring, which makes it possible to tell the methylated DNA fragments and the unmethylated targets apart only by a pair of specific primers [15]. Unlike MSP, which can only be used for qualitative detection, both qMSP and MS-HRM can quantitatively detect the level of DNA methylation through simple PCR reaction and instrumental analysis. Comparing with pyrosequencing, MS-HRM can not only detect a wide range of methylation levels but also help researchers and technicians save more time and cost for each test so as to become a more economical method that can be used more widely [12]. MS-HRM can be used to make up for the imprecision and high demands of qMSP since it has a higher detection sensitivity. All these make MS-HRM to be a high-efficiency and cost-effective detection method, especially suitable for the basic laboratory to carry out.

## 2. Materials and Methods

### 2.1. Extraction of Tissue Sample DNA and Bisulfite Modification

A total of 57 formalin-fixed, paraffin-embedded (FFPE) tissue samples were collected from EMPD patients admitted to Huashan Hospital; 23 FFPE normal tissues and 31 other types of cancer tissues were obtained from the Department of Pathology in Huashan Hospital North. This study has been approved by the institutional review board of Huashan Hospital, and all patients have provided their written informed consent. All these samples were processed for DNA extraction using QIAamp DNA FFPE Tissue Kit (Qiagen, Germany) followed by bisulfite modification using the EZ DNA Methylation-Gold Kit (Zymo Research, USA) as described in our previous study [8].

### 2.2. Methylated Standard Preparation

The Universal Methylated Human DNA Standard was purchased from Zymo Research (Cat no. D5011). Both standards (100% methylated and 0% methylated DNA) were treated with bisulfite by EZ DNA Methylation-Gold Kit (Zymo Research, USA). The concentration of standards was primarily measured by real-time PCR on an ABI PRISM 7500 Fast Sequence Detection System (Applied Biosystems, USA) to get the Ct value of each sample. Ct values were used to calculate the mixing ratio of 50% methylated DNA. According to the preliminary ratio measured by real-time PCR, a series of approximative mixing ratios was tried to make several possible 50% methylated DNA samples. All these samples subsequently went through pyrosequencing in the pyrosequencer PyroMark Q24 (Qiagen) to determine the exact mixing proportions. A series of concentration gradients of methylated positive control DNA was constructed at the ratios of 100%, 50%, 30%, 10%, 5%, 1%, and 0%. The detailed introduction of pyrosequencing is listed in the subsequent part.

### 2.3. MS-HRM

The primers for MS-HRM were designed according to the guideline of Wojdacz et al. [16], which targeted the CpG islands in the *MSH*2 promoter: forward: 5′-CGTAGTTTTGGAAGTTGATTGGGT-3′; reverse: 5′-CGAAACCTCCTCACCTCCTAATT-3′.

A total of 20 *μ*l mix was prepared to undergo a PCR: 10 *μ*l DNA Polymerase Premix (PrimeSTAR HS DNA Polymerase Premix, Takara), 0.8 *μ*l for each forward and reverse primer (10 *μ*M, Jie Li Biology), 1 *μ*l SYTO9 (SYTO™ 9 Green Fluorescent Nucleic Acid Stain, Invitrogen), 0.8 *μ*l modified DNA sample, and 6.6 *μ*l ddH_2_O. After thoroughly mixed, DNA samples were amplified in a thermal cycler (Veriti™ 96-Well Thermal Cycler, Applied Biosystems) observing the following conditions: 95°C for 10 min, 40 cycles for 95°C for 10 s, 60°C for 10 s, 72°C for 10 s, ending with 72°C for 5 min.

The PCR products were transferred to a Rotor-Gene Q 5plex HRM Platform (Qiagen) to conduct HRM analyses using the following conditions: hold 1 : 95°C for 1 min, hold 2 : 40°C for 2 min, HRM: 65–95°C, rising by 0.1 degree each step, and hold 3 : 35°C for 1 min.

In every experiment, the unknown samples were analyzed by MS-HRM together with seven methylated standards at different methylation levels. After the amplification and detection, the normalized fluorescence values were recorded. By comparing the average fluorescence values of unknown samples at 83.5°C, 84.5°C, 85.5°C, and 86.5°C to the MS-HRM standard curve, the proximate percentage of methylation levels can be calculated. All tissue samples detected by MS-HRM were independently conducted three times, and the results listed in the following sections were the average values of three tests.

### 2.4. Pyrosequencing

Pyrosequencing of the *MSH*2 promoter was performed on PyroMark Q24 (Qiagen), and the detailed operation procedures were the same as described in our previous study [8]. First of all, bisulfite-modified DNA samples were amplified using HotStarTaq DNA Polymerase Premix (Takara) with the following primers: forward primer: 5′-biotin-TTTGGAAGTTGATTGGGT-3′ and reverse primer: 5′-ACCTCCTCACCTCCTAATTAA-3′. After amplification, PCR products were processed with PyroMark Gold Q24 Reagents (Qiagen) and then sequenced on PyroMark Q24 according to manufacturer's instructions using a sequencing primer: 5′-ACCCACTAAACTATTTCC-3′.

### 2.5. Statistical Analysis

MS-HRM standard curve was analyzed using Rotor-Gene 6000 Series Software. Pearson's correlation test and Bland–Altman analysis were employed to compare the correlation coefficient between MS-HRM and pyrosequencing using GraphPad Prism 7 software.

## 3. Results

### 3.1. Construction of the MS-HRM Standard Curve to Detect the Methylation Level of the *MSH*2 Gene Promoter

**Figure 1:**
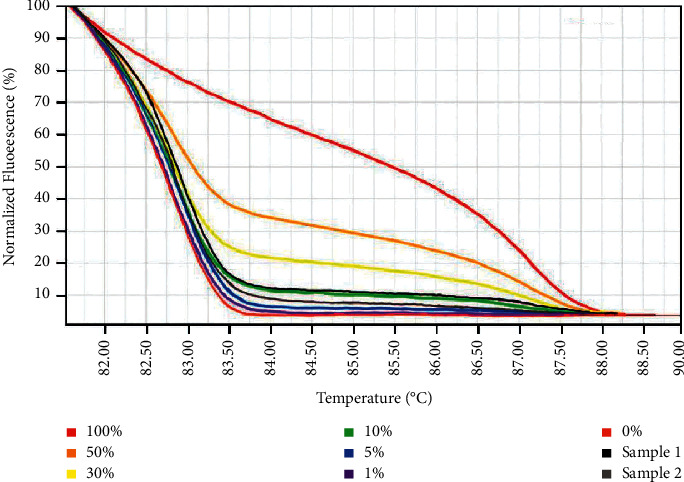
Using the MS-HRM standard curve to detect the methylation level of the *MSH*2 gene promoter. Standard curves are shown, from top to bottom: 100%, 50%, 30%, 10%, 5%, 1%, and 0% of standard methylated DNA. The lines in black and grey are two example results of EMPD samples.

For rapid and accurate detection of *MSH*2 methylation, a MS-HRM standard curve was constructed by using a series of methylated positive control DNA with the concentration gradients of 100%, 50%, 30%, 10%, 5%, 1%, and 0%. EMPD samples modified by bisulfite were simultaneously amplified with the standard curve in every independent test, and the methylation level of *MSH*2 can be determined using this MS-HRM standard curve (Figure 1). In this study, a total of 57 EMPD samples were analyzed, and 96.5% (55/57) samples had hypermethylation in the *MSH*2 promoter.

Besides, 23 normal controls and 31 samples of other cancer types were also detected by MS-HRM with the same standard curves in this study including 4 normal skin tissues, 19 nontumor adjacent tissues, 19 colorectal cancer (CRC) tissue samples, 5 breast cancer tissues, 4 bladder cancer tissues, 1 thyroid cancer tissue, 1 kidney cancer tissue, and 1 endometrial cancer tissue. Among all these samples, two CRC tissue samples showed positive *MSH*2 methylation with 1% and 5%, respectively. Our results indicated that the primers designed in this experiment for MS-HRM are widely applicable to various diseases.

### 3.2. Comparison of MS-HRM and the Pyrosequencing Method on the Profile of *MSH*2 Methylation

**Figure 2:**
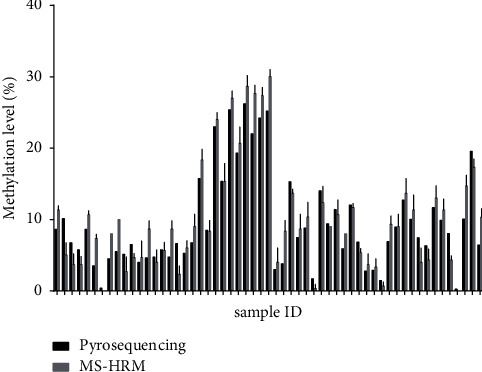
Profile of *MSH*2 methylation (%) in 57 EMPD samples with both MS-HRM and pyrosequencing techniques.

**Figure 3:**
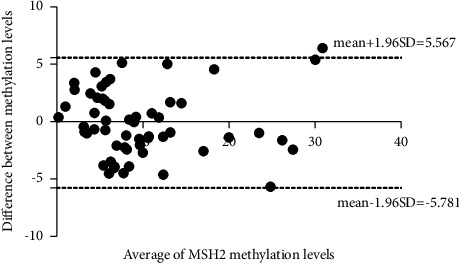
Bland–Altman plot analysis: each point represents a sample, the horizontal axis represents its average value, and the vertical axis indicates the difference between the two methods. The 95% limits of agreement were adopted and marked as dotted lines.

To verify the accuracy of the results detected by MS-HRM, pyrosequencing technique was employed to test all EMPD samples as a reference method. The results of both methods are summarized in Figure 2. Pearson's correlation test (Table 1) indicated a high correlation between the methylation results of MS-HRM and pyrosequencing (*r* = 0.9425, *p* < 0.0001). By Bland–Altman plot analysis, all samples except one (sample 29) were within the 95% limits of agreement indicating there was no difference between these two techniques (Figure 3). The Bland–Altman plot showed the mean bias ± 1.96 SD between the *MSH*2 methylation levels detected by pyrosequencing and MS-HRM assay as −0.1069 ± 5.674 (%), and the limits of agreement were −5.781 and 5.567. For sample 29, the result of MS-HRM was higher than that of pyrosequencing. Although the above analysis confirmed that these two approaches show a high degree of consistency, the methylation levels obtained from MS-HRM were slightly higher than from pyrosequencing in 59.6% of samples (34/57).

## 4. Discussion

MMR plays a key role in maintaining the fidelity of DNA replication and genome stability. Impaired MMR pathway typically results in tandem repeat sequences known as microsatellite instability (MSI) and promotes tumorigenesis [17]. MMR deficiency is strongly related to the development of multiple cancers, such as colorectal cancer (CRC), endometrial carcinoma (EC), and breast cancer, as well as glioma and lymphoma [18, 19]. For those patients with Lynch syndrome with high-frequency MSI, about 50% might possibly have tumors other than CRC/EC, including urothelial, prostate, and pancreas tumors, according to Latham et al.'s study published in 2019 [20].

DNA methylation can work as the initial force to drive mutagenesis, and the level of DNA methylation is strongly related to different statuses of diseases [21, 22]. Methylation modification of MMR genes may be responsible for the absence of MMR protein expression and MSI in most cases. In MMR-deficient ECs, 76% of cases were caused by DNA methylation, and those patients with both MMR deficiency and methylation inactivation were closely related to larger tumor size and lower disease-specific survival [23]. In CRC patients, 12% of MSI was caused by methylation of MMR genes, *MLH*1 in particular, which led to subsequent silencing of genes [24]. Kahn and his colleagues found out that, in a total of 5917 patients with endometrial cancer, 1044 patients were tested *MLH*1 immunohistochemistry (IHC) absent, of which 86.3% were considered to be due to the presence of DNA methylation in *MLH*1 [25]. The study above also found that 40% of patients with Lynch syndrome harbored *MSH*2 mutations which were much higher than *MLH*1 mutations (16%). However, this study did not detect the methylation level of *MSH*2 [25]. In our study, 10% (2/19) of CRC tissue samples were tested methylation positive in the *MSH*2 promoter which led to a decrease in *MSH*2 expression. CRC patients with pathogenic germline *MSH*2 mutations were more likely to develop somatic *MSH*2 hypermethylation, which suggests a potential linkage between germline *MSH*2 mutations and hypermethylation of this gene [26].

Unlike the in-depth studies of MMR in CRC, there are relatively limited research studies exploring MMR inactivation and methylation in skin carcinogenesis so far. However, some reports have showed the evidence of the correlation between MMR deficiency and spontaneous skin tumors [27, 28]. In Merkel cell carcinoma (MCC), for instance, 10.9% of MCC patients had impaired *MSH*2 expression, while 16.1% of patients had reduced expression in at least one MMR protein [29]. According to Vasan et al.'s report [30], about 9.1% of cutaneous head and neck squamous cell carcinomas were defective in MMR expression. For patients with Muir–Torre syndrome (MTS), *MSH*2 was the most frequently mutated MMR gene, and about 90% of the MMR gene mutations in MTS were found in *MSH*2 [31, 32]. Immunohistochemistry test of MMR and detection of MMR deficiency might be used as a potential tool to assist the diagnosis of MTS [33].

Currently, certain methods are available to detect the DNA methylation status of specific genes [34]. Sequencing-based approach and PCR-based assays are the most common methods used in methylation detection. Pyrosequencing is capable of precise quantification, but it is laborious and time-consuming. Besides, it has higher requirements not only for expensive equipment but also for well-qualified operators. So, in this research, we focus on PCR-based methods which are suitable for most primary hospitals and clinical laboratories. Among the three most commonly used PCR-based methods, MSP can only perform qualitative analysis via a gel electrophoresis, while qMSP and MS-HRM provide quantitative detection. The result of MS-HRM can be interpreted directly according to the standard curves eliminating the trouble of complex analysis, and it is appropriate for most regions, while qMSP is more suitable for CpG-rich regions, and it requires two sets of specific primers which put forward extremely stringent requirements on primer design and amplification efficiency [12, 35]. In view of its certain advantages, we use the MS-HRM approach as an optimal tool in this research.

Quantitative detection of abnormal MMR methylation is valuable for disease diagnosis, treatment monitoring, and research. In the treatment of cancers, MMR serves the prediction of the potential benefit from chemotherapy. The National Comprehensive Cancer Network (NCCN) has officially included the assessment of MMR into the guideline of CRC in 2018. Clinical evidence has shown that defected MMR status in CRC patients as well as gastric cancer (GC) patients may render 5-fluorouracil (5-FU) based chemotherapy ineffective [36, 37]. In muscle-invasive bladder cancer, *MSH*2 deficiency resulted in the resistance to cisplatin and brought about poorer patient survival showing *MSH*2 can potentially serve as a biomarker predictive of response to platinum-based therapy [38]. Moreover, recent evidence revealed that deactivated MMR led to active neoantigen generation which may provide a new approach to developing novel targeted therapies [39]. Enhanced immune surveillance due to MMR inactivation provides an additional opportunity for immunotherapy, of which programmed death-1 (PD-1) inhibitor is more efficient and effective in MMR-deficient CRC patients [40]. Thus, reduced MMR capacity makes a contribution to a promising immune checkpoint blockade therapy [41]. All these studies showed the potential value of *MSH*2 detection in tumor treatment. MS-HRM, as a reliable and accurate method, is suitable for the quantitative analysis of *MSH*2 methylation to aid the diagnosis and treatment of these diseases.

In this study, we established and provided a fast method to assess *MSH*2 methylation levels by MS-HRM. Through this efficient, economical, and convenient tool, *MSH*2 status of all EMPD samples was evaluated effectively, and the test results correlated highly with pyrosequencing. The validity of MS-HRM standard curves was confirmed in our research. The primers and the whole system can be used in both EMPD samples and other cancer types which may better facilitate the diagnosis and therapeutic regimen. Besides, the detection rate of *MSH*2 methylation was much higher than our previous results (73.6%, 42/57) in the same samples by performing methylation-specific PCR (MSP) [8]. Since MSP can only be used to detect the methylated CpGs designed in the specific primers, therefore, the sensitivity of MS-HRM is considered higher than MSP. It is also difficult to visually distinguish extremely faint electrophoretic bands which may lead to false negative results. However, the sample size in this research was limited, and a larger cohort should be tested for validation to enhance the effectiveness of this study. In conclusion and based on our results, it appears feasible and worth attempting MS-HRM for the detection of *MSH*2 promoter methylation.

## Figures and Tables

**Table 1:** Coefficient of correlation between MS-HRM and pyrosequencing methylation results.

Parameters	*MSH*2 gene
Variable *Y*	MS-HRM
Variable *X*	Pyrosequencing
Sample size	57
Correlation coefficient *r*	0.9425
Significance level	<0.0001
95% confidence interval for *r*	0.9038 to 0.9658

## Data Availability

The data of MS-HRM detection and pyrosequencing used to support the findings of this study are included within the article.
